# Efficacy of continuous positive airway pressure in neonates with respiratory distress syndrome: A protocol for systematic review and meta-analysis

**DOI:** 10.1097/MD.0000000000026406

**Published:** 2021-06-18

**Authors:** Junyi Cao, Zuowu Chen, Jinbing You, Jiangjiang Wang, Qiongyao Tang

**Affiliations:** a Department of Pediatrics, The First People's Hospital of Jiangxia District; b Department of Pediatrics, Hubei Maternal and Child Health Care Hospital, Wuhan 430200, Hubei, P. R. China.

**Keywords:** continuous positive airway pressure, efficacy, neonates, respiratory distress syndrome

## Abstract

**Background:**

Respiratory distress syndrome (RDS) is a condition caused by a deficiency in pulmonary surfactant. Many interventions, including pulmonary surfactant, non-invasive respiratory support, and other supportive treatments have been used to prevent RDS. However, recent studies have focused on the continuous positive airway pressure as a significant potential agent for preventing RDS. However, its safety and effectiveness are yet to be assessed. To this end, the current study aims to perform to explore the safety and effectiveness of continuous positive airways in treating neonates with RDS.

**Methods:**

We will conduct comprehensive literature searches on MEDLINE, Cochrane Library, EMBASE, Chinese National Knowledge Infrastructure, and Chinese BioMedical Literature from their inception to April 2021. The search aims to identify all the randomized controlled studies on continuous positive airway pressure in treating neonates with RDS. In addition, we aim to search the gray literature to establish any available potential studies. We will use 2 independent authors to determine study eligibility, extract data using the structured pro-forma table, analyze data, and utilize suitable tools in assessing the risk of bias in the selected studies. Accordingly, we will conduct all statistical analyses using RevMan 5.3 software.

**Results:**

The current study aims to provide high-quality synthesis of existing evidence concerning the continuous positive airway pressure to treat neonates suffering from RDS.

**Conclusion:**

Our findings seek to provide evidence to establish whether continuous positive airway pressure can ascertain safety and effectiveness for neonates with RDS.

**Ethics and dissemination:**

The study will require ethical approval.

**OSF registration number:**

May 20, 2021.osf.io/7nj8s. (https://osf.io/7nj8s/).

## 1 Introduction

Respiratory distress syndrome (RDS) is instigated by a deficiency or dysfunction of pulmonary surfactant. The surfactant lines the alveolar surface, preventing atelectasis at end-expiration. Accordingly, a pulmonary surfactant is considered an active agent capable of keeping the pulmonary alveoli open as well as facilitating the entry of air to the lungs to enhance oxygenation in neonates.^[1,2]^ In particular, the disease is mainly found in preterm newborns, characterized by a progressive intensification in respiratory effort and a reduction in the amount of air entering the lungs, hence favoring hypoxia.^[3]^ Accordingly, the physiologic role of surfactant entails the capacity to lower surface tension. It also has the capacity of rapidly absorbing, spreading, and reforming a monolayer in the dynamic conditions related to the respiratory cycle.^[4]^ At the same time, the administration of exogenous surfactant can moderate mortality and risk of air leak, especially since it is regarded as the basis of therapy for preterm infants with RDS.^[5,6]^

In the recent past, non-invasive respiratory support has become more and more prevalent for respiratory dysfunction management, especially among preterm newborns.^[7,8]^ Drawing from existing studies, it is evident that application of nasal continuous positive airway pressure from birth is considered effective as intubation and ventilation for newborns, especially those that are less than 30 weeks growth.^[9–11]^ However, applying continuous positive airway pressure from the beginning in an unselected population of preterm newborns can cause the risk of under-treating of those infants RDS and for those whom continuous positive airway pressure might fail to afford sufficient respiratory support. At the same time, the effectiveness of continuous positive airway pressure is well-known extensively acceptable among clinicians; however, whether patients benefit more or less from continuous positive airway pressure is less clear. Still, continuous positive airway pressure has been approved to be used for neonates with RDS, meaning that it is considered crucial to understanding the efficacy and safety of continuous positive airway pressure to treat neonates with RDS. Therefore, our study aims to evaluate the safety and effectiveness of continuous positive airway pressure in treating neonates with RDS.

## 2 Objectives

The present study seeks to explore the safety and effectiveness of continuous positive airway pressure in treating neonates suffering from RDS.

## 3 Methods

### 3.1 Study registration

The present protocol was registered in OSF (https://osf.io/) under number 10.17605/OSF.IO/7NJ8S. It follows the guidelines put forward by Preferred Reporting Items for Systematic Review and Meta-Analysis Protocol statement.^[12]^

### 3.2 Criteria for considering studies

#### 3.2.1 Types of studies

Our study will consider the use of a randomized or quasi-randomized controlled trial to investigate the safety and effectiveness of continuous positive airway pressure in treating neonates with RDS.

#### 3.2.2 Types of participants

The study intends to include premature newborns (less than 37 weeks gestation), particularly those considered to be at risk of developing RDS of those with an already existing clinical diagnosis of RDS.

#### 3.2.3 Types of interventions

We will include continuous positive airway pressure treatment with any pressure level. We will also consider those delivered by any type of device, for any given duration, and presented in any mode, compared to no-continuous positive airway pressure or sham continuous positive airway pressure. We will also include studies that applied continuous positive airway pressure at any time after the patient presentation.

#### 3.2.4 Types of outcome measures

The major outcomes for the present study are the number of neonates needing mechanical ventilation as well as recovery time. However, the minor outcomes will include changes in the respiratory rate, change in the saturation of the arterial oxygen, emergency department stay duration, hospital stay duration, the requirement for admission in the intensive care unit, and other adverse incidents.

### 3.3 Search methods for identification of studies

We will carry out an in-depth literature search on MEDLINE, Cochrane Library, EMBASE, Chinese National Knowledge Infrastructure, and Chinese BioMedical Literature from their inception to April 2021, to identify all the randomized controlled studies on continuous positive airway pressure in treating neonates suffering from RDS. We will employ the following search terms singly or as combinations: “respiratory distress syndrome,” “continuous positive airway pressure,” and “randomized controlled trial.” Besides, we intend to search the gray literature to identify any potential studies.

### 3.4 Data collection and analysis

#### 3.4.1 Selection of studies

**Figure 1:**
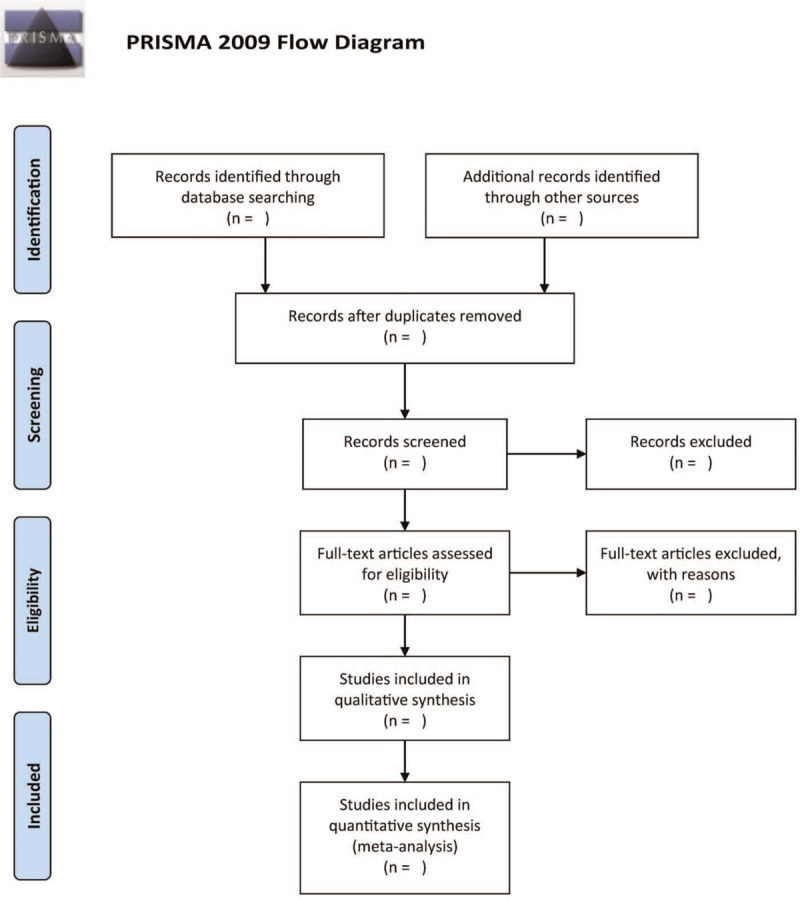
The research flowchart.

We intend to include all randomized and quasi-randomized controlled trials that will meet the established selection criteria prescribed in the previous section. Accordingly, we will use 2 independent authors to review the search results and independently select studies that meet the inclusion criteria. Also, we will resolve any disagreements between the authors through discussion and by involving a third author. Figure 1 illustrates the study selection process.

#### 3.4.2 Data extraction and management

We will use 2 independent authors to also extract, assess, and code all data obtained from the search, for every study, through the use of a pilot data extraction designed purposefully for our study. Also, we plan to utilize a third author to resolve any disagreements between the 2 independent authors. Also, the authors will independently enter data into RevMan 5.3, with another author checking the entries.

#### 3.4.3 Assessment of risk of bias in included studies

Furthermore, we plan to employ 2 independent authors to evaluate the risk of bias of all included trials by utilizing the Cochrane “Risk of bias” tool.^[13]^ In case of disagreements, we intend to resolve them through discussion with a third author.

#### 3.4.4 Measures of treatment effect

We will analyze continuous outcome data using the mean differences (MDs) or standardized MDs. Also, we will analyze dichotomous outcome data by utilizing the relative risk. In particular, we intend to report the 95% confidence intervals on all estimations.

#### 3.4.5 Dealing with missing data

We will rely upon evaluators to verify key study features and attain missing numerical outcome data where pertinent.

#### 3.4.6 Assessment of heterogeneity

Our study will also rely on utilizing the *I*^*2*^ statistic in measuring the heterogeneity of the studies in every analysis. Where we find significant heterogeneity (*P* < .1, and *I*^*2*^ > 50%), we intend to use the random-effects models; otherwise, we will use the fixed-effects model.^[14,15]^

#### 3.4.7 Assessment of reporting biases

Our study will also consider creating and examining a funnel plot to examine possible small studies as well as publication biases where more than 10 studies are included.

#### 3.4.8 Sensitivity analysis

Furthermore, our study will perform sensitivity analysis to investigate our findings’ reliability and robustness.

## 4 Discussion

The present study aimed at assessing the safety and effectiveness of continuous positive airway pressure in treating neonates with RDS. Recently, the randomized controlled trials of continuous positive airway pressure for the treatment of neonates suffering from RDS have increased gradually. While many published studies seem to suggest that the application of continuous positive airway pressure has a significant position to treat newborns suffering from RDS, its safety and effectivity in treating neonates with RDS is still indecisive. Therefore, our study seeks to explore this topic, assessing the safety and effectivity of continuous positive airway pressure in treating neonates with RDS. We are confident that our study presents a practical guide for practitioners to make valid decisions when treating neonates with RDS. Our study will also be critical for health policymakers.

## Author contributions

**Conceptualization:** Junyi Cao, Zuowu Chen.

**Data curation:** Junyi Cao, Zuowu Chen, Jinbing You.

**Formal analysis:** Junyi Cao, Jinbing You.

**Funding acquisition:** Jiangjiang Wang, Qiong-Yao Tang.

**Investigation:** Jiangjiang Wang.

**Methodology:** Junyi Cao, Jinbing You, Jiangjiang Wang.

**Project administration:** Jiangjiang Wang, Qiong-Yao Tang.

**Resources:** Junyi Cao, Jiangjiang Wang, Qiong-Yao Tang.

**Software:** Junyi Cao, Zuowu Chen.

**Supervision:** Jinbing You, Jiangjiang Wang.

**Validation:** Zuowu Chen.

**Visualization:** Zuowu Chen.

**Writing – original draft:** Junyi Cao, Zuowu Chen.

**Writing – review & editing:** Jiangjiang Wang, Qiong-Yao Tang.
